# Lesion-induced insights in the plasticity of the insect auditory system

**DOI:** 10.3389/fphys.2013.00048

**Published:** 2013-08-23

**Authors:** Reinhard Lakes-Harlan

**Affiliations:** AG Integrative Sensory Physiology, Institute for Animal Physiology, Justus-Liebig-University GießenGießen, Germany

**Keywords:** auditory systems, plasticity, regeneration, axotomy

## Abstract

The auditory networks of Orthoptera offer a model system uniquely suited to the study of neuronal connectivity and lesion-dependent neural plasticity. Monaural animals, following the permanent removal of one ear in nymphs or adults, adjust their auditory pathways by collateral sprouting of afferents and deafferented interneurons which connect to neurons on the contralateral side. Transient lesion of the auditory nerve allows us to study regeneration as well as plasticity processes. After crushing the peripheral auditory nerve, the lesioned afferents regrow and re-establish new synaptic connections which are relevant for auditory behavior. During this process collateral sprouting occurs in the central nervous networks, too. Interestingly, after regeneration a changed neuronal network will be maintained. These paradigms are now been used to analyze molecular mechanism in neuronal plasticity on the level of single neurons and small networks.

Like other neural systems in insects, auditory networks have long been regarded as inflexible neural systems. A main basis for this impression is that in some taxa, like Orthoptera, auditory systems are involved in intraspecific communication whose signals are relatively constant in a population. These acoustic signals facilitate species discrimination which implies reliable neuronal processing of such signals. Orthopteran auditory systems are divers, but they have in common that about 20–80 primary sensory neurons are located in the peripheral ear. The sensory axons project ipsilaterally into a target neuropile in the respective ganglia of the central nervous system (CNS; Figure 1; Stumpner and von Helversen, 2001). Within the neuropile the afferents are monosynaptically connected to individually identifiable first order interneurons. These interneurons process and transmit the information either to the contralateral side or to higher centers in the CNS. The different auditory systems are quite well-known in their anatomy, physiology, and behavior in intact animals, allowing to analyze lesion-induced neuronal plasticity. Two different experiment approaches have been used. Firstly, the information transfer has been interrupted permanently with a removal of one ear. Anatomical, physiological and behavioral analyses show astonishing neuronal plasticity in the auditory system. Secondly, a transient lesion, an axotomy of the auditory afferents has been used to study a combination of regenerative and plasticity processes. Here the results of lesions in auditory systems of Orthoptera are reviewed in respect to the two paradigms and in respect to the first data on molecular mechanisms behind the processes.

**Figure 1:**
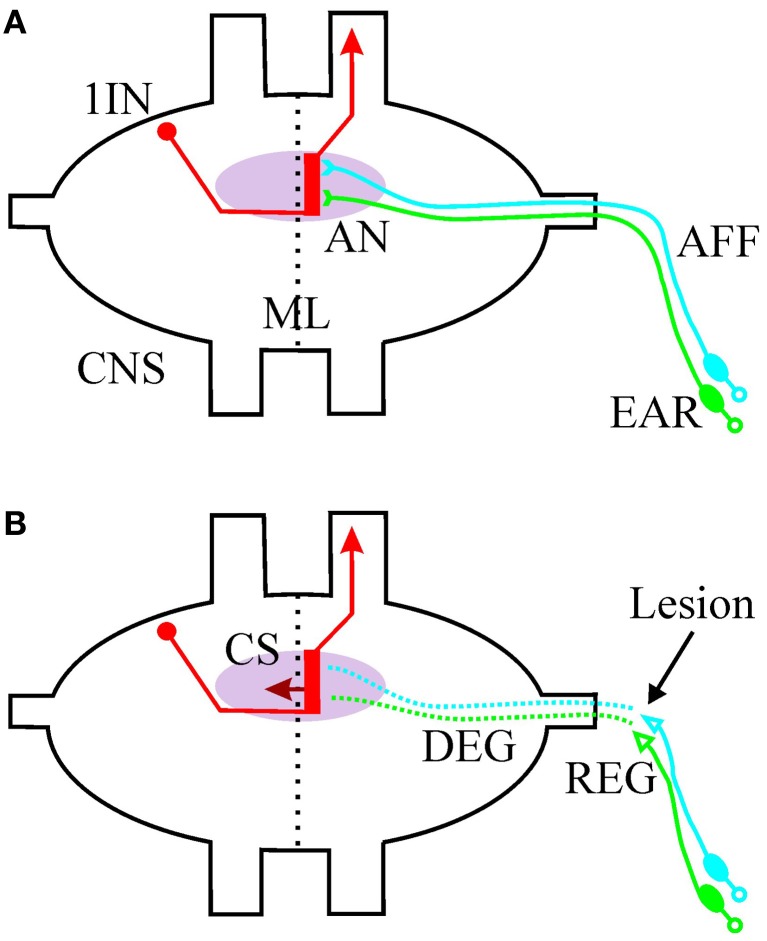
**Schematic drawing of the afferent auditory system of Orthoptera (exemplified for Ensifera). (A)** The primary sensory neurons are located in the peripheral ear and project their axons (AFF, afferents) to an auditory neuropile (AN, pink) in the central nervous system (CNS). The afferents synapse onto the dendritic region (red bar) of first order auditory interneurons (1IN). The processing is strictly ipsilateral of the midline (ML). The interneurons could transport the information to the contralateral side (not shown) and/or toward higher centers in the CNS. **(B)** After a peripheral lesion of the afferents (arrow), the distal part of the axon degenerates (DEG) and regeneration starts at the proximal part (REG). First order interneurons grow collateral sprouts (CS) across the midline into the intact part of the auditory neuropile.

## Permanent lesion

Adult insects cannot regenerate lost organs. Therefore a loss of an ear with its sensory cells results in a permanent interruption of auditory afferent activity on one side of the CNS. Consequently, an asymmetry of information processing takes place which, for example, influences the directionality of the auditory system in the CNS. In many Orthoptera, phonotactic behavior can be used for analysis of changes and plasticity in auditory system. During phonotaxis monaural individuals typically move toward the intact side (side of highest excitation), resulting in circling instead of a linear approach to a sound source. Nevertheless, these insects might reach the sound source, as has been documented for crickets and bush crickets (Huber, 1987; Lakes and Mücke, 1989; Schmitz, 1989). It could even be shown that the directionality might improve with time, despite an ongoing changed excitation balance in the nervous system (Schmitz, 1989). Such changes are clearly indicative for plasticity processes in the afferent pathways of adult insects. During this process, interneurons connected monosynaptically to afferents might sprout collaterals and establish new synaptic connections (Schildberger et al., 1986; Schildberger and Kleindienst, 1989; Schmitz, 1989; Lakes et al., 1990). Interestingly, these interneurons typically sprout into the contralateral intact neuropil, although how the process is initiated and how the target is recognized remains poorly understood. Recent work has identified first molecular factors during this process and it could be shown that the guidance molecule *sema2a* is upregulated during dendritic growth (Horch et al., 2009, 2011). Collateral sprouts can also cross the midline, whereas in embryonic development regulatory sequences direct fiber growth across the midline. It will be interesting to see if and how factors like *slit* or *robo* are involved in controlling adult neuronal morphology and collateral sprouting after lesion. So far, neither in the adult locust nor in the Drosophila CNS an expression of *slit* or *robo* could be immuncytochemically detected up to 48 h after lesion (Lakes-Harlan et al., unpublished). Perhaps the factors are important only during embryonic development, raising the question how collateral sprouting is regulated in adults.

Typically only interneurons monosynaptically connected to the lesioned afferents react with a sprouting. Why do only deafferented interneurons react (and to a small part contralateral auditory afferents)? Molecular cues from degenerating afferents will be present for all neurons in the neuropile and adjacent areas. Neurotrophic factors comparable to vertebrates are not known in insects, so it will be interesting to analyse anterograde or retrograde signals at insect synapses. Due to the permanent removal of one ear, the corresponding neuropile shrinks in volume (Krüger and Lakes-Harlan, 2010). The neurochemical composition of the neuropile area, however, is largely unchanged. The histochemical staining intensity for acetylcholinesterase is decreased by less than 10%, whereas with immunocytochemistry no change was found for γ-amino butyric acid and serotonin (Krüger and Lakes-Harlan, 2010). The synapse specific vesicle-associated membrane protein (*vamp*) is downregulated 7 days after deafferentation (Horch et al., 2011).

A lost ear is not completely regenerated even if the lesion happened in the first larval instar. Crickets have a rather good regeneration capacity of peripheral structures; however, even in these insects a complete and functional regeneration of the complex hearing organ has never been demonstrated (Huber, 1987). Crickets which have regenerated a seemingly entire leg will still only regenerate parts of the auditory system, like tympanal membranes and a few sensory units. Hearing is always impaired due to the incomplete rebuilding (Huber, 1987). Beside the complexity of the sensory organ, the failure of functional regeneration might also be due to the type of sensory cell. Auditory receptors of insects belong to scolopidial sensory units. Each unit consists typically of a sensory cell, a glial cell, an attachment cell, and a scolopidial cell. The scolopidial cell forms the characteristic scolopale, which surrounds the sensory dendrite. Although these scolopidia share many developmental pathways with other peripheral sensory sensilla, they regenerate differently. Hair sensilla embedded in other regenerating epidermal tissue regenerate rather easily (Lüdke and Lakes-Harlan, 2008). By contrast scolopidial units forming complex sensory organs are only rarely regenerated in Orthoptera. These differences in regeneration of sensory neurons are not understood so far, but might be related to general regenerative capabilities. In phasmids, which have a huge regeneration capability of appendages, scolopidial organs are regenerated inside the legs (Friedrich, 1930).

## Transient lesion

A transient lesion in the auditory afferents starts regenerative processes in addition to possible plastic processes in the central auditory network. A typical transient lesion is an axotomy, performed either as tympanal nerve crush or as nerve cut. No mayor differences in respect to regeneration capacity between crushed and cut axons have been reported in insects. Many studies use nerve crush as an experimental paradigm, because a crushing usually leaves the nerve sheath intact and the sheath might function as a guiding structure. Such operations have been performed on the tympanal nerve in different species of Orthoptera (Pallas and Hoy, 1988; Jacobs and Lakes-Harlan, 2000) and in all cases a neuronal regeneration of the sensory axons occurred. The axotomy did not elicit an immediate change in the electrical response properties of the axons, although some minutes after the operation the number of action potentials per acoustic stimulus might increase (Lakes-Harlan, 2004). The axotomy divides the neuronal fiber in two parts: the proximal part remains intact and a Wallerian degeneration back to the cell bodies has not been observed in insects. The distal part of the auditory axon (i.e., the central arborization) that is separated from the cell body degenerates (Figure 1B). Such degeneration in not self-evident as isolated axons in insects may survive disconnected from the cell body for a long time. In very small insect species anucleate processes might even be regular parts of neuronal networks (Polilov, 2012). In the CNS of locusts a fragmentation, but no degeneration, of lesioned axons by glial cells has been observed (Jacobs and Lakes-Harlan, unpublished). By contrast, in the peripheral auditory system the degeneration has been demonstrated ultrastructurally (Jacobs and Lakes-Harlan, 1999). Glial cells are involved in the degeneration process, and markers for glial cells, like glial cell binding lectins, show a temporary increased binding in the auditory neuropil (Jacobs and Lakes-Harlan, 1997). The degeneration takes about 3–5 days, but the afferent pathway to the CNS and within the CNS might contain degeneration products and an increased number of glial cells processes for a much longer time. Therefore, the axonal pathway is likely to contain a distinct molecular signature that might be used for guided regeneration.

The proximal part of the axon and the cell soma can start regenerative growth (Figure 1B). Probably not all, but a significant number of sensory neurons regenerate neuronal processes after a crush in both, adults and juveniles, respectively. Shifts in the hearing threshold curves during regeneration indicate that not all fibers regenerate (Krüger et al., 2011a). The initial signal for regrow is not yet determined in insects. Immunohistochemically, an upregulation of the transcription factor p53 expression, but not of CREB (cAMP response element-binding protein) could be shown in sensory neurons after tympanalnerve lesion in *Schistocerca gregaria* (Sohn and Lakes-Harlan, unpublished). In the brain of *Drosophila* factors like c-Jun N-terminal protein kinase are involved in axonal regeneration and target recognition (Ayaz et al., 2008).

At the tip of the regenerating nerve, actin and tubulin seems to be upregulated (evidenced by immunhistochemistry on lesioned tympanal nerves of adult *Schistocerca gregaria*; Sohn and Lakes-Harlan, unpublished). The next step during regeneration involves neuronal pathfinding. Regenerating axons often follow the original paths; however, they might leave these pathways or use other pathways from the beginning. This indicates that degeneration products are not necessary for pathfinding, although they might support it. In sensory systems a key guidance molecule could be the homophilic cell adhesion molecule Fasciclin I. This cell surface molecule is expressed on probably all sensory axons of insects during embryogenesis (Bastiani et al., 1987); its expression is maintained in the adult nervous system in distinct sensory neuropil areas, but not in others (Jacobs and Lakes-Harlan, 2000). Upregulation of this molecule has been demonstrated in neuronal regeneration in the antennal system, but not yet for auditory axons (Stern et al., 2012). However, it has been documented that regenerating auditory afferents can follow Fasciclin I positive non-auditory axonal tracts (Jacobs and Lakes-Harlan, 2000). But eventually, such misled auditory afferents leave the false tracts and reach the correct target area. The regenerated fibers arborize only within the target auditory neuropile. The molecular cues for recognizing the target area and for the development of specific terminal arborizations are not known. Typically a regenerated axonal projection shows some anatomical plasticity as it has distinct features, like longer collaterals, discriminating it from a normal projection. In many cases the projection area is enlarged, as proven for regenerated fibers from hair sensilla on the locust leg (Lüdke and Lakes-Harlan, 2008). Interestingly, the regenerated projection is independent from lesions on contralateral afferents (Krüger and Lakes-Harlan, 2011). Such double lesion experiments were designed in order to detect factors influencing the projection pattern. First an axotomy was performed on one side and later the contralateral ear was removed. However, this secondary deafferentation of the auditory neuropile did not provide cues strong enough to trigger the ipsilateral regenerated fibers to cross the midline or to show a different regeneration pattern (Krüger and Lakes-Harlan, 2011), despite the fact that deafferentation can modulate afferent sprouts in crickets (Horch et al., 2011).

A question often raised in regeneration process is, whether developmental processes are recapitulated. In embryos of locusts the pathfinding of auditory afferents has been described, and in contrast to regeneration, no irregular fiber growth (like a trial- and-error growth) on the way to the target area could be found (Schäffer and Lakes-Harlan, 2001). During embryonic development the afferents as well as the target area express Fasciclin I which might be important for axonal guidance. Thus, the processes during development and regeneration are different.

The regenerated fibers synapse onto auditory interneurons in the target neuropil. Such regenerated synapses are functionally relevant for auditory information processing; however, not all behavioral functions are restored. In the grasshopper *Chorthippus biguttulus* regenerated fibers contribute to the discrimination of the direction of auditory signals, but not to the recognition of species specific sounds (Lakes-Harlan and Pfahlert, 1995). For a directed phonotactic reaction the animals have to recognize the species specific sound signal *and* the direction. For the sound recognition an intact ear was always necessary; after lesions on both ears, recognition of species specific sounds failed. The same lack of complete functional recovery has recently been observed in the tettigoniid *Mecopoda elongata* (Friedrich and Lakes-Harlan, unpublished). Either the regenerated synaptic connections are not specific enough, or a plasticity reaction to the transient deafferentation has changed the neuronal networks permanently. Perhaps even a combination of both possibilities takes place during regeneration. It is likely that interneurons of the network form aberrant connections in response to the lesion (Figure 1B). These modified connections are based on collateral sprouting and have been shown in various species (Pallas and Hoy, 1988; Lakes and Kalmring, 1991; Krüger et al., 2011a). Additionally, evidence has been found that these connections do not re-change to normal after reinnervation. Compensatory sprouting is independent from the stage of the operation and for physiological compensation of deafferentation no age related differences between nymphs and adults could be found (Krüger et al., 2011b).

Sprouting of auditory interneurons and regeneration of afferent shows that growth is possible in the CNS. However, as in vertebrates regenerative growth capabilities in insects might be different in the peripheral nervous system and in the CNS. The environment in the peripheral nerve allows regeneration of sensory axons in all investigated cases. In the CNS, interneuronal axonal regeneration could be shown in the locust embryo (Pätschke et al., 2004; Stern and Bicker, 2008), but in other cases little regeneration capacities have been found (Ayaz et al., 2008). However, insects have started to provide model systems for neuronal regeneration and plasticity, as factors like erythropoietin, nitrogen monoxide, VAMP and c-jun N-terminal protein kinase could be found to influence or regulate these processes (Ayaz et al., 2008; Stern and Bicker, 2008; Ostrowski et al., 2011).

## Outlook

Recent results show the immense capacities of neuronal growth, target recognition, synapse formation, and compensatory plasticity after lesion in the auditory system of insects. The auditory system could offer new insights into plasticity, as the neuronal networks are rather well-known and lesions can be easily performed. Physiological and behavioral plasticity can be investigated with different experimental paradigms. With the further advent of molecular tools, regulatory mechanisms in the CNS will be unraveled in the locust or cricket. Approaches like differential display PCR which already indicated that regenerating tympanal sensory neurons in locusts express other genes than control neurons (Jacobs, 1997) are needed in the future. Insects provide an important platform for such studies, especially as neuronal regeneration is generally not suppressed by inhibitory factors, like myelin in mammals. Nevertheless, insect neurons normally grow during development and seem to remain stable in the adult. A lesion, however, might trigger collateral growth. What are the decisive factors which influence the balance between dynamic growth and stable morphology? The identified neurons of the auditory system are likely to offer a suitable tool to answer this question in the near future.

### Conflict of interest statement

The author declares that the research was conducted in the absence of any commercial or financial relationships that could be construed as a potential conflict of interest.
